# Pseudotumor in metal-on-metal hip arthroplasty: a comparison study of three grading systems with MRI

**DOI:** 10.1007/s00256-018-2873-0

**Published:** 2018-02-01

**Authors:** C. Smeekes, B. J. M. Schouten, M. Nix, B. F. Ongkiehong, R. Wolterbeek, B. C. H. van der Wal, R. G. H. H. Nelissen

**Affiliations:** 1 Department of Orthopaedics, Leiden University Medical Centre, 9600, 2300, RC, Leiden, The Netherlands; 2 Department of Orthopaedics, Meander Medical Centre, Amersfoort, The Netherlands; 3 Department of Radiology & Nuclear Medicine, Maasstad Hospital, Rotterdam, The Netherlands; 4 Department of Radiology, Meander Medical Centre, Amersfoort, The Netherlands; 5 Department of Medical Statistics and Bioinformatics, Leiden University Medical Centre, Leiden, The Netherlands

**Keywords:** THA, Pseudotumor, Classification, MARS-MRI, MoM, Grading system, Interobserver reliability

## Abstract

**Objective:**

Pseudotumors, a well-known complication of metal-on-metal total hip arthroplasty (MoM THA), are well identified on metal artifact-reducing sequences magnetic resonance imaging (MARS-MRI). Several MRI grading systems are described in the orthopedic literature, but their validity is unknown in large clinical studies. Our study was undertaken to describe the classification of pseudotumors in a preselected cohort divided into high- and low-risk patients, using three pseudotumor grading systems applied on MARS-MRI, and to determine the interobserver reliability of the grading systems.

**Patients and methods:**

A retrospective study was performed on 377 consecutive patients (240 MRI scans) treated with an M2a-38 and Taperloc stem combination (Biomet, Warsaw, IN, USA). Patients were divided into a high-risk and a low-risk group based on previous published risk factors. Two observers determined the presence of pseudotumors using three different pseudotumor grading systems for classifying MARS-MRI results.

**Results:**

The prevalence of pseudotumors as determined with MARS-MRI was 59% in our high-risk group, 0% in the low-risk group and 43% in the control group. Serum cobalt values were increased in the high-risk group. The kappa values of the Anderson, Hauptfleisch and Matthies grading system scores were 0.43, 0.44, and 0.49 respectively.

**Conclusions:**

High-risk patients are at a high risk for pseudotumor development. No pseudotumor development was found in low-risk patients. Interobserver reliability scored best with the Matthies system, but all three grading systems showed only a moderate agreement.

## Introduction

Concerns have been raised on the use of metal-on-metal total hip arthroplasty (MoM THA) because of frequent early revision rates [1], raised cobalt and chrome serum levels in the blood and their possible toxicological effects [2], and the occurrence of cystic and/or solid massed, or so-called pseudotumors, in the periprosthetic tissue [3]. Recent studies show that the incidence of pseudotumors is comparable with other THA systems such as cobalt on polyethylene and metal on polyethylene [4, 5]. MoM THA-induced pseudotumors can cause compression of the neurovascular bundle [6, 7]. This had also been described for conventional THA, although this pseudotumor compression was associated with a broken or worn-out inlay, which induces a MoM reaction [8, 9]. A possible explanation could be that the MoM reaction stimulates the formation of pseudotumors with a larger mass than those observed in conventional THA, although recent research rejected this suggestion [4]. Associations between the presence of a pseudotumor and serum cobalt levels have been described [10]. High local cobalt values may induce pseudotumor formation and are known to cause osteolysis [11]. Risk factors for the formation of a pseudotumor are cobalt >5 μg/l [10], female gender [12, 13], pain [14], and a high inclination angle >55° [15]. Despite the observed associations and risk factors, the exact mechanism of THA-induced pseudotumors is still unclear. Pseudotumors are well defined on MRI and three grading systems have been described in the orthopedic literature by Anderson [16], Matthies [17], and Hauptfleisch [3]. Table 1 provides details of the scoring systems.

**Table 1:** Comparison of the three grading systems

Grading system	Features	
Anderson [1]		
A Normal	Normal post-operative appearances including seromas and small hematomas	
B Infection	Fluid-filled cavity with high-signal T2 wall; inflammatory changes in soft tissues; ± bone marrow edema	
C1 Mild MoM	Periprosthetic soft-tissue mass with no hyperintense T2-weighted fluid signal or fluid-filled peri-prosthetic cavity; either less than a maximum of 5 cm in diameter	
C2 Moderate MoM	Periprosthetic soft-tissue mass/fluid-filled cavity greater than 5 cm in diameter or C1 lesion with either of the following: muscle atrophy or edema in any muscle other than short external rotators, or bone marrow edema: hyperintense on STIR	
C3 Severe MoM	Any of the following: fluid-filled cavity extending through deep fascia, a tendon avulsion, intermediate T1weighted soft-tissue cortical or marrow signal, fracture	
Hauptfleisch [2]		
Type 1	Thin-walled cystic mass (cyst wall <3 mm)	
Type 2	Thick-walled cystic mass (cyst wall >3 mm, but less than the diameter of the cystic component)	
Type 3	A predominantly solid mass	
Matthies [3]		
1	Thin-walled	Fluid-like: hypointense on T1, hyperintense on T2 shape flat, with walls mainly in apposition
2a	Thick-walled	Fluid-like: hypointense on T1, hyperintense on T2 shape not flat
	Or irregular	>50% of the walls not in apposition
2b	Thick-walled	Atypical fluid: hyperintense on T1, variable on T2 any shape
	Or irregular	
3	Solid throughout	Mixed signal, any shape
Other findings scored by the radiologists		
The dimensions of the pseudotumor		
Gluteus minimus muscle atrophy		
Gluteus medius muscle atrophy		
Presence of a fluid-filled bursa		
Soft-tissue erosion (extension of the pseudotumor into the surrounding muscles)		
Bone marrow edema		
Tendon tears		

Van der Weegen et al. [18] described kappa values for all three classification systems (49 hips) and Chang et al. [14] describes a kappa for the Anderson classification (192 hips). Importantly, other radiological studies on pseudotumors do not report a kappa value and therefore the results should be interpreted with caution [3, 19–21]. The reproducibility of these grading systems is of clinical importance and may help to unravel the etiology of a pseudotumor.

The aims of this study were to describe the classification of pseudotumors in a preselected cohort by utilizing metal artifact-reducing sequences magnetic resonance imaging (MARS-MRI) in a high-risk and low-risk group for pseudotumor development, and to study the interobserver reliability of three different pseudotumor grading systems in a large single cohort of MoM THA (240 hips).

## Patients and methods

Our investigation reviewed 377 uncemented MoM THA performed in our institution between February 2008 and January 2011. In all cases, a cobalt and chromium bearing couple consisting of a monoblock acetabular cup with a 38-mm fixed size head design was implanted (M2a-38; Biomet, Warsaw, IN, USA). The cup size ranged from 48 mm to 64 mm. All patients received a press-fit titanium femoral stem (Taperloc, Biomet, Warsaw, IN, USA).

All patients were subjected to a pre-defined screening protocol, which was initiated after the first concerns of the MoM THA. The clinical results of this screening were reported recently [22]. In the current study, all patients with an MoM THA and available MARS-MRI were selected.

### Screening protocol

Patients received a standardized outpatient consultation. This included physical examination, patient-reported questionnaires, blood analysis for serum cobalt, radiographs of the hip and pelvis, and magnetic resonance imaging (MRI). Contrast-enhanced MRI of the hip region with MARS was performed in patients with osteolysis on the X-ray, elevated serum metal ion levels above 5 μg/l, or pain. Patients without these criteria received routine annual follow-up, which was the same as the first screening.

Pain was defined as the presence or absence of any pain in the hip area reported by the patient.

Cobalt and chromium ion levels were determined in the serum with the use of an AAnalyst 800 Atomic Absorption Spectrophotometer (Perkin Elmer, Waltham, MA, USA). The blood samples were collected in a metal-free container. Serum cobalt levels ranging from 0.04 to 0.64 μg/l are considered normal in the general population [23]. Cobalt serum levels higher than 5 μg/l were defined as elevated in MoM THA [2]. Inclination was measured on the 6-week post-operative X-ray. The observer who performed the measurements had shown good reliability for measuring post-operative cup inclination angle (ICC = 0.74,* p* = <0.001) in a previous study (Smeekes et al., accepted for publication)

Patients were divided into three groups based on the likelihood of developing a pseudotumor: one group was supposed to have a high risk for developing a pseudotumor based on the literature. Selection criteria for the high-risk group were: serum cobalt >5 μg/l [10], female sex [12, 13], hip pain [14] and a high cup inclination angle >45°, which is considered to be out of the safe zone in resurfacing prosthesis [16, 24]. Inclusion criteria for the low-risk group were the opposing criteria (male sex, no hip pain, cup inclination <45°, and serum cobalt <5 μg/l). All other patients were used as the control group.

For a description of the pseudotumors, the Anderson classification [16], the classification of Matthies [17], and the Hauptfleisch classification were used [3]. Also, a list of other findings on the MRI scan that are not reported in these scores is shown (Table 1). An experienced musculoskeletal radiologist and a musculoskeletal radiologist in training independently scored the MRI scans. The radiologists applied all grading systems at the same time. Kappa values were compared for each grading system. Discordant cases were discussed and consensus was obtained for the classification system with the best kappa values.

For the MRI scans, an MRI scanner with a field strength of 1.5 Tesla was used. The following MARS-MRI sequences were used: T1-weighted coronal plane with echo time (TE) 16 ms, repetition time (TR) 450–650 ms, slices (SL) 2.5 mm, field-of-view (FOV) 36 × 37.1, and bandwidth (BW) 435 Hz/pixel; T2-weighted coronal plane with TE 80 ms, TR 3,500–7,000 ms, SL 2.5 mm, FOV 36 × 45, and BW 435 Hz/pixel; T2-weighted short tau inversion recovery (STIR) coronal plane with TE 40 ms, TR shortest ms, SL 3.5 mm, FOV 36 × 45, and BW 435.5 Hz/pixel.

The scientific committee of the Leiden University Medical Centre and the ethics committee in the Meander Medical Centre waived ethical approval.

### Statistical analyses

Descriptive analyses were performed on baseline data and final outcomes. The results are expressed as means with standard deviations or medians with ranges where relevant. Reliability of the radiological measurements was evaluated by calculating interclass correlation coefficients using Cohen’s kappa. The differences among the cobalt values in the pseudotumor classification group were analyzed using post-hoc tests in a one-way ANOVA after logarithmic transformation of the cobalt values because of the skewed (positive) distribution of these values. Chi-squared test was used for the analyses of gender and pain between the pseudotumor classifications. Fisher’s test was used to analyze the difference between the presence of a pseudotumor in the high- and low-risk groups. A* t* test was used for comparing the serum cobalt levels in patients in the control group with and without a pseudotumor. For all tests, a two-tailed significance level of 0.05 was used. SPSS software (version 20; IBM, Armonk, NY, USA) was used for the analyses.

## Results

A total of 240 patients with an M2a-38 and Taperloc stem combination (Biomet, Warsaw, IN, USA) were eligible for analyses. Demographics and reason for surgery are listed in Table 2. Seventy-five patients had bilateral prostheses: all 75 had prostheses of the M2a-38 type with a Taperloc stem and were included in the study. The contralateral hip prostheses were of different types and were not included in the study. Twenty-three patients had a bilateral M2a-38 prosthesis and Taperloc stem (=46 M2a-38) combination and 119 patients had a unilateral MoM prosthesis of the M2a-38 type and a Taperloc stem (=119 M2a-38).

**Table 2:** Demographics, indications for surgery, and cobalt serum values of the high- and low-risk patients

Demographics	Total (*n* = 240)	High risk group (*n* = 34)	Low risk group (*n* = 5)	Control group (*n* = 201)
Age in years, mean (SD)	63.2 (7.4)	62.9 (5.6)	61.5 (3.0)	63.3 (7.8)
Follow-up in months, mean (SD)	29.6 (10.0)	31.4 (9.7)	29.8 (8.1)	29.3 (10.1)
BMI, kg/m^2^, mean (SD)	27.4 (4.3)	28.5 (4.2)	25.7 (6.1)	27.1 (4.2)
Gender,* n* (%)				
Male	79 (33)	0 (0)	5 (100)	74 (36.8)
Female	161 (67)	34 (100)	0 (0)	127 (73.2)
Side,* n* (%)				
Left	99 (41)	12 (35.3)	4 (80)	83 (41.3)
Right	141 (59)	22 (64.7)	1 (20)	118 (58.7)
Bilateral THA^a^	75	16	0	59
Bilateral M2a-38^b^	23 (46)	5 (10)	0	18 (36)
Unilateral M2a-38	119	8	5	106
Indication,* n* (%)				
Osteoarthritis	218 (90.8)	32 (94.1)	4 (80)	182 (90.5)
Secondary osteoarthritis	9 (3.8)	1 (2.9)	0 (0)	8 (4.0)
Collum fracture	9 (3.8)	1 (2.9)	1 (20)	7 (3.5)
Osteonecrosis of the head	4 (1.7)	0 (0)	0 (0)	4 (2.0)
Cobalt serum level, mean (SD)	9.5 (15.0)	19.8 (34.6)	1.6 (0.9)	8.0 (7.1)

^a^Bilateral THA indicates a M2a-38 metal-on-metal total hip arthroplasty on one side and a different type of hip prosthesis on the contralateral side

^b^Bilateral M2a-38 indicates a M2a-38 metal-on-metal total hip arthroplasty on both sides

### Classification of the pseudotumors

**Fig. 1:**
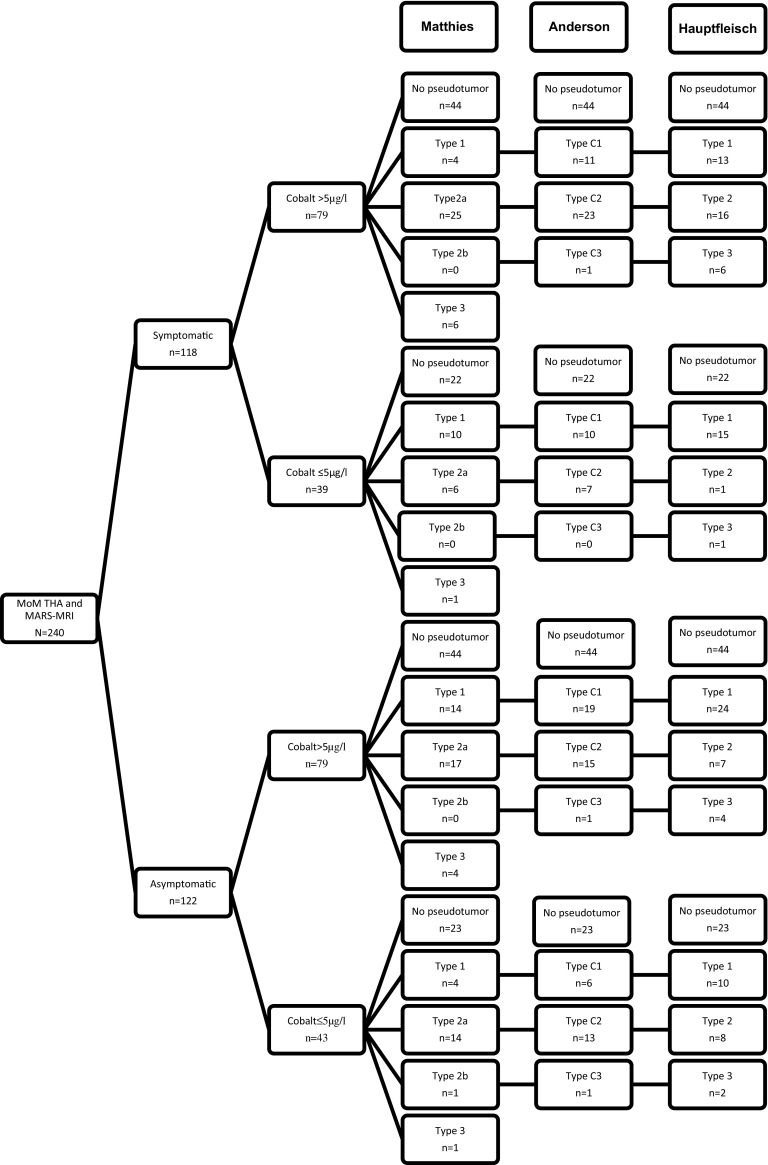
Flowchart of patients, observer 1. (Anderson type b was not represented because there were no scores).* MoM THA* metal-on-metal total hip arthroplasty,* MARS-MRI* metal artifact-reducing sequences magnetic resonance imaging

**Fig. 2:**
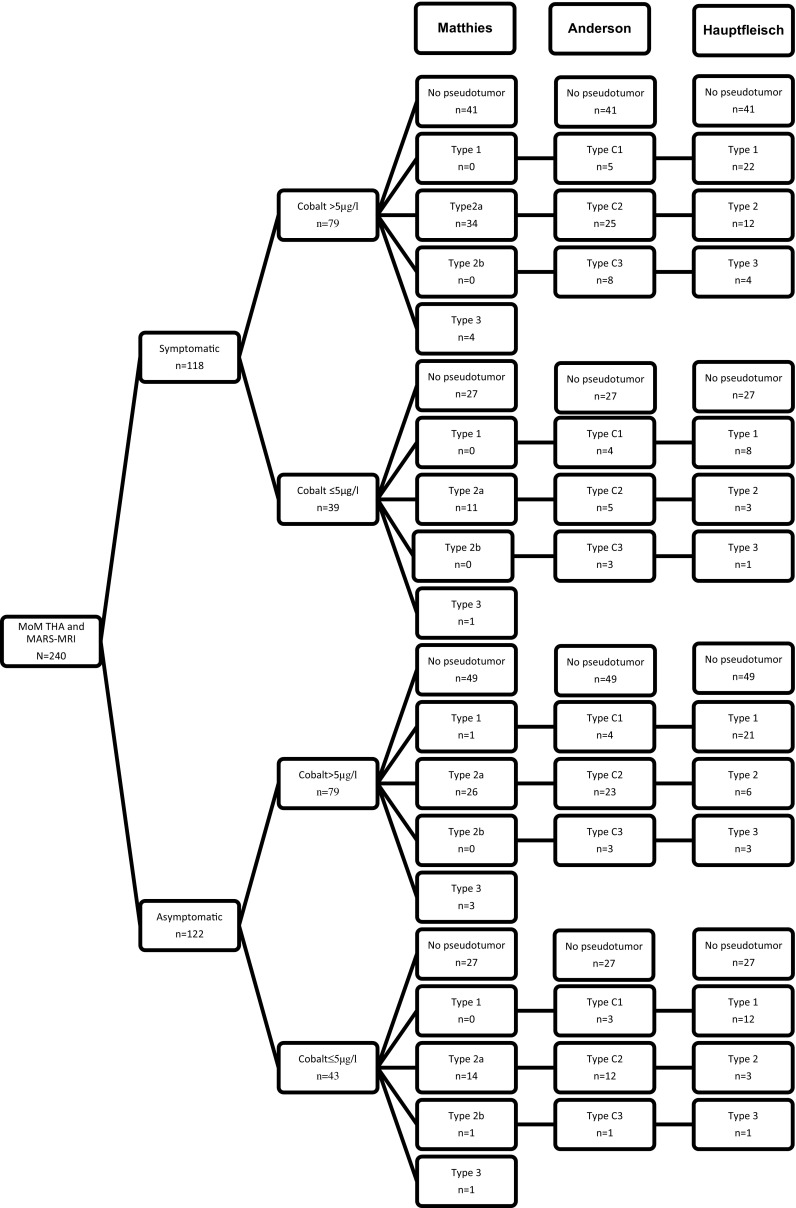
Flowchart of patients, observer 2. (Anderson type b was not represented because there were no scores)

In this single cohort of MoM THA hips, observer 1 identified 107 pseudotumors (44.6%) and observer 2 identified 96 pseudotumors (40%) regardless of the grading system used. Observer 1 identified: 46 type C1, 58 type C2, and 3 type C3 pseudotumors using the Anderson grading system. Using the Matthies grading system, observer 1 identified: 32 type 1, 62 type 2a, 1 type 2b, and 12 type 3 pseudotumors. With the use of the Hauptfleisch grading, 62 type 1, 32 type 2, and 13 type 3 pseudotumors were classified. (Fig. 1; Table 3) Observer 2 identified: 16 type C1, 65 type C2, and 15 type C3 pseudotumors using the Anderson grading system. Using the Matthies grading system observer 2 identified 1 type 1, 85 type 2a, 1 type 2b, and 9 type 3 pseudotumors. With the use of the Hauptfleisch grading, 63 type 1, 24 type 2, and 9 type 3 pseudotumors were classified by observer 2 (Fig. 2; Table 3). Interobserver reliability on whether a pseudotumor was present or not was 0.56 (*p* = <0.001). Interobserver reliability for pseudotumor severity with the Anderson, Matthies, and Hauptfleisch grading systems was 0.43 (*p* = <0.001), 0.49 (*p* = <0.001), and 0.44 (*p* = <0.001) respectively. In our cohort, the Matthies score was the most reliable classification (Table 4).

**Table 3:** Findings on MRI by two observers

Findings on MRI	Observer 1	Observer 2
Pseudotumor		
Yes	107	96
No	133	144
Anderson		
C1	46	16
C2	58	65
C3	3	15
Hauptfleisch		
1	62	63
2	32	24
3	13	9
Matthies		
1	32	1
2a	62	85
2b	1	1
3	12	9
Dimensions of the pseudotumor		
Height in mm, mean (SD)	26.4 (39.5)	31.1 (45.0)
Width in mm, mean (SD)	17.1 (25.8)	22.2 (31.9)
Wall thickness in mm, mean (SD)	0.5 (0.75)	0.5 (0.69)
Other findings		
Gluteus minimus muscle atrophy	90	39
Gluteus medius muscle atrophy	72	16
Presence of a fluid-filled bursa	62	58
Soft-tissue erosion	37	28
Bone marrow edema	12	4
Tendon tears	8	2

**Table 4:** Interclass correlation (ICC) of the findings on MRI

ICC	Kappa	*p* value
Presence of a pseudotumor	0.56	<0.001
Classification		
Anderson	0.43	<0.001
Hauptfleisch	0.44	<0.001
Matthies	0.49	<0.001
Other findings		
Gluteus minimus muscle atrophy	0.26	<0.001
Gluteus medius muscle atrophy	0.33	<0.001
Presence of a fluid-filled bursa	0.6	<0.001
Soft-tissue erosion	0.59	<0.001
Bone marrow edema	0.29	<0.001
Tendon tears	0.19	<0.001
Foci of susceptibility	0.45	<0.001

A 17% complete agreement between observer 1 and observer 2 was reached for Anderson C1, 68% for Anderson C2, and 6% for Anderson C3. For the Matthies system, 3% complete agreement between observer 1 and observer 2 was reached for grade 1, 73% for grade 2a, 100% for grade 2b, and 75% for grade 3. For the Hauptfleisch system, 48% complete agreement between observer 1 and observer 2 was reached for grade 1, 53% for grade 2, and 39% for grade 3.

The Matthies grading system was discordant for the results of 70 scans. Observer 1 scored 21, no pseudotumors, whereas observer 2 scored the same scans as follows: 1 grade 1, 18 grade 2a, and 2 grade 3 pseudotumors. Observer 1 scored 32 scans as a grade 1 pseudotumor and observer 2 scored 23 as having no pseudotumor, whereas only 9 were scored as grade 1 pseudotumor. Observer 1 scored 10 grade 2a pseudotumors where observer 2 scored 8 no pseudotumors and 2 grade 3 pseudotumors. Lastly, observer 1 scored 7 scans as a grade 3 pseudotumor whereas observer 2 scored 1 no pseudotumor and 6 grade 2a pseudotumors.

A bursa filled with fluid (without connection with the joint) was found for 25.8% cases by observer 1 and for 24.2% of the cases by observer 2. Atrophy of the gluteus medius and minimus muscles was scored by observer 1 in 90 cases, whereas observer 2 scored 39 cases as positive. Atrophy of the gluteus medius muscle was found in 72 of the cases by observer 1 and in 16 cases by observer 2.

**Fig. 3:**
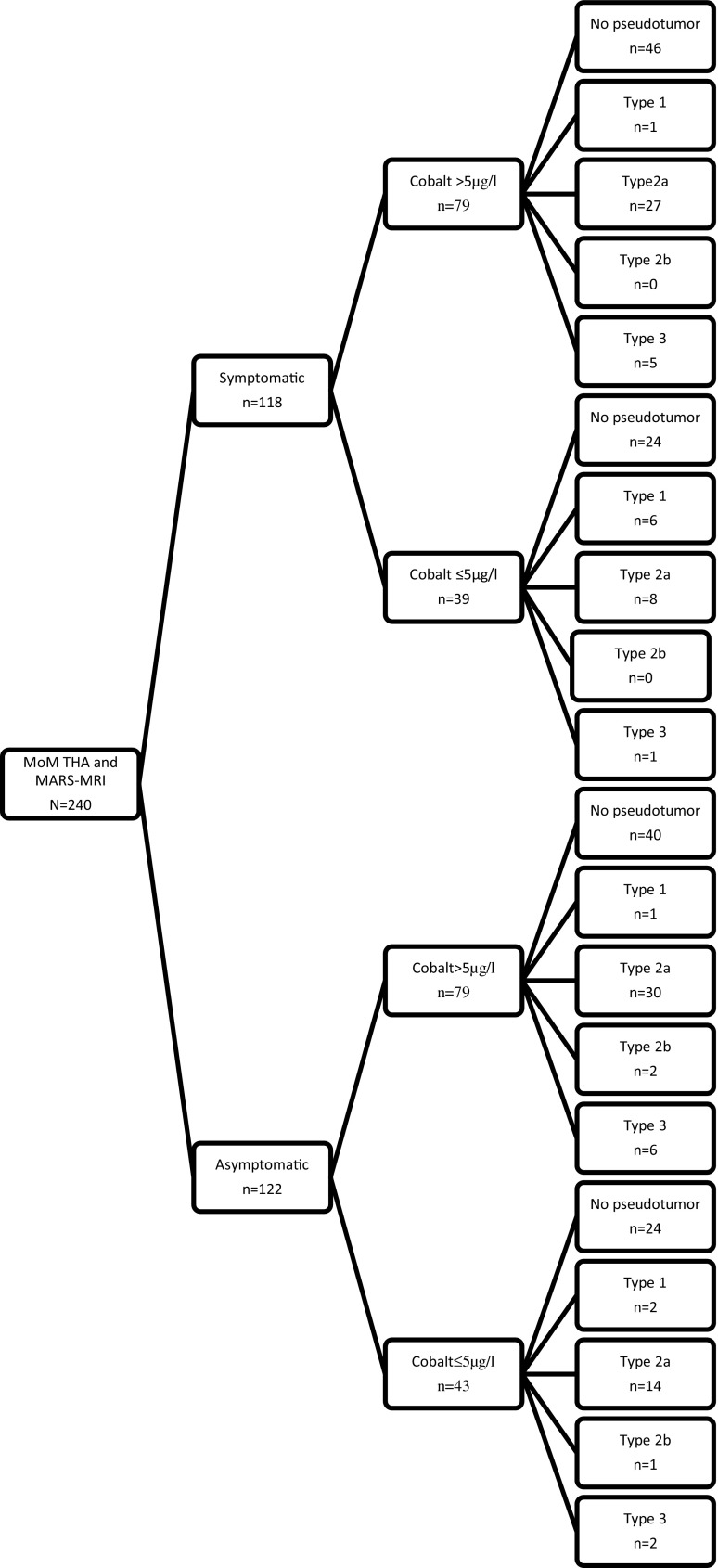
Flowchart of patients after consensus on the Matthies classification

After the consensus, 106 pseudotumors were diagnosed with the use of the Matthies grading system. Type 2a pseudotumor was the most frequent classification (Fig. 3). Patients with a type 2a pseudotumor had a mean serum cobalt of 13.1 μg/l (SD 23.7). Thirty-eight of these patients had pain and 61 were female. After the logarithmic transformation of the cobalt values, a significant difference could be observed between the group of patients without a pseudotumor and the group of patients with a type 1 or 2a pseudotumor (*p* < 0.05), and also between a type 1 pseudotumor versus a type 2a pseudotumor (*p* < 0.05) or type 3 pseudotumor (*p* < 0.05) respectively. No difference between the pseudotumor classification and pain or gender could be detected (Table 5). Of the 106 pseudotumors, 52 were asymptomatic (49%). There was no difference between the presence of a pseudotumor in the symptomatic patients (54 out of 118) compared with the asymptomatic patients (52 out of 122; *p* = 0.62; Table 6). Regarding categories of pseudotumor wall thickness (no wall, <3 mm or ≥3 mm), there were no differences between the symptomatic and asymptomatic patients (Table 5).

**Table 5:** Classification of pseudotumors after consensus of the observers with the differences between the classification groups

Matthies classification	No pseudotumor (*n* = 134)	Type 1 (*n* = 10)	Type 2a (*n* = 79)	Type 2b, (*n* = 3)	Type 3 (*n* = 14)
Mean serum cobalt (SD)	7.8 (7.3)	4.0 (5.1)	13.1 (23.7)	8.4 (6.1)	10.2 (15.0)
Male/female	84/50	6/4	18/61	1/2	4/10
Pain	64	7	38	2	7

There are significant differences between the group of patients without a pseudotumor versus the group of patients with a type 1 (*p* < 0.05) or 2a (*p* < 0.05) pseudotumor respectively, and of type 1 versus type 2a (*p* < 0.05) or versus type 3 (*p* < 0.05) respectively

**Table 6:** Pseudotumors in symptomatic and asymptomatic patients. Based on consensus assessments

Pseudotumor	Yes (%)	No (%)	Total (%)
Symptomatic	54 (45.8)	64 (54.2)	118 (100)
Asymptomatic	52 (42.6)	70 (57.4)	122 (100)

### High- and low-risk patient screening

After classification of pseudotumors according to the Matthies grading, patients were divided in a high-risk group for pseudotumor development, a low-risk group, and a control group. A significantly higher risk was found for pseudotumor development in the high-risk group (59%, 20 out of 34) versus the low-risk group (0%, 0 out of 5; *p* < 0.001). In the control group, 86 pseudotumors were diagnosed in 201 THAs (43%). The patients in the control group with a pseudotumor had a mean serum cobalt of 8.3 μg/l (SD 6.6 μg/l) and patients in the control group without a pseudotumor had a mean serum cobalt level of 7.8 μg/l (SD 7.5 μg/l; *p* = 0.61).

A difference was found between cobalt serum values and high- and low-risk patients. No differences were found in the control group between patients with a pseudotumor and those without a pseudotumor with regard to serum cobalt levels. No significant differences in the type of pseudotumor, pain, and gender were found among the groups. Also, no differences in the control group were found between symptomatic and asymptomatic patients.

## Discussion

As far as we know, this is one of the largest series of MoM THA in which patients were screened for having a pseudotumor by utilizing MARS-MRI [25]. A total of 106 pseudotumors were diagnosed after consensus in 240 MoM THAs (44%), of which 49% had no symptoms. In the low-risk patient group (male sex, no hip pain, cup inclination <45°, and serum cobalt <5 μg/l), no pseudotumors were diagnosed, whereas a high percentage of pseudotumors (59%, 20 out of 34) was found in the high-risk patient group (serum cobalt >5 μg/l [10], female sex, hip pain, and a high cup inclination angle >45°). However, the control group also showed a high percentage of pseudotumors (43%, 86 out of 201). The high-risk group showed a significantly higher risk of developing a pseudotumor.

A higher cobalt level is also correlated with different levels of pseudotumors. One may hypothesize that a (local) higher cobalt level influences the formation of a pseudotumor; however, several studies show that cobalt values are a poor predictor [17, 26, 27]. In the literature, it is reported that the existence of a pseudotumor varies by type of prosthesis. In large-head MoM prosthesis patients, 40–60% of the patients develop a pseudotumor [10, 28, 29]. Our cohort results agree to these findings. Other studies showed that the prevalence of pseudotumors in conventional THA ceramics on polyethylene and metal on polyethylene is comparable with MoM THA [4, 5], but they are less symptomatic and revisions due to pseudotumors are rare in non-MoM THA. More patients with MoM THA report pain compared with patients with conventional THA [30], which may be caused by the local toxicity of the cobalt, hypersensitivity reactions on the metal release, subsequent osteolysis, and soft-tissue damage [22, 31]. Chang et al. reports that soft-tissue damage is associated with pain and not the presence or size of a pseudotumor [14]. The behavior of pseudotumors in the long term is unclear.

A high complication rate (14%) in MoM revision surgery has been reported, with a 7% re-revision rate after 2 years (range: 26–52 months) [32] and a dislocation rate of up to 28% [33–35]. Three of the studies used a posterior approach and one study used a posterior approach in 80% of the cases and in 20% an anterolateral approach. The exact reason for the higher luxation rate after these revisions is not clear, but all authors suggest that the extensive destruction of soft tissue caused by the MoM prosthesis might play an important role. Atrophy of the gluteal musculature and subsequent instability may contribute to the high dislocation rate. In the case of symptomatic patients, MARS-MRI can be used as a preoperative tool to classify the damage of the soft tissue before revision and add to decision-making to choose a dual mobility cup to lower the dislocation rate [36].

To the best of our knowledge, only one study has reported the interobserver reliability among the three grading systems [18]. In this study, the highest kappa (0.58) was found for the Anderson grading system. In our study, the highest kappa (0.49) was found for the Matthies classification, 0.44 for the Anderson classification, and 0.43 for the Hauptfleisch classification. Although this study included a larger cohort of patients compared with van der Weegen et al., a lower kappa value was measured. We do not have an explanation for this, but in both studies, there was only moderate agreement, which questions the reproducibility and thus clinical use. Another difference is that we only used the M2a-38 system and in the other study, three different systems had been used. A shortcoming of the study is that the MARS-MRI scored all grading systems at the same time, which can create a “cross-contamination” of results. The low kappa value of the Anderson score can be partly explained by the inclusion of more parameters of periprosthetic tissue compared with the Matthies and Hauptfleisch scores, which only score the characteristics of the pseudotumor.

Compression by a pseudotumor on the neurovascular bundle, soft-tissue damage, and osteolysis aid in the clinical decision-making whether to revise or not.

In conclusion, a higher occurrence of pseudotumor development in high-risk patients was found. No pseudotumor development was found in low-risk patients. However, patients in the control group also showed a high occurrence of pseudotumors. This means that every patient, except those defined as “low risk,” is at a substantial risk for developing a pseudotumor. No differences were found in the control group between patients with a pseudotumor and those without a pseudotumor with regard to serum cobalt levels. The Matthies score was the most reliable classification, but all three grading systems showed limited interobserver reliability. MARS-MRI is one of the tools that aids in clinical decision-making regarding revision MoM THA, but whether or not a pseudotumor grading system should be used, or if so, which one, is still under debate.
